# Magnitudes of Anemia and Its Determinant Factors Among Lactating Mothers in East African Countries: Using the Generalized Mixed-Effect Model

**DOI:** 10.3389/fnut.2021.667466

**Published:** 2021-07-28

**Authors:** Biruk Shalmeno Tusa, Adisu Birhanu Weldesenbet, Nebiyu Bahiru, Daniel Berhanie Enyew

**Affiliations:** ^1^Epidemiology and Biostatistics Department, College of Health and Medical Sciences, Haramaya University, Haramaya, Ethiopia; ^2^Department of Public Health and Health Policy, School of Public Health, College of Health and Medical Sciences, Haramaya University, Haramaya, Ethiopia

**Keywords:** anemia, lactating mother, Eastern Africa, demographic and health survey data, generalized mixed effect model

## Abstract

**Background:** The number of studies on the magnitude of anemia and its determinant factors among lactating mothers is limited in East African countries regardless of its multivariate consequences. Even though few studies were conducted on the magnitude of anemia and its determinants, most of them focused on the country level and different parts of countries. Therefore, the current study is aimed to determine the magnitude of anemia and determinant factors among lactating mothers in East African countries.

**Methods:** From nine East African countries, a total weighted sample of 25,425 lactating mothers was included in the study. Determinate factors of anemia were identified using generalized linear mixed models (GLMM). Variables with a *p* < 0.05 in the final GLMM model were stated to confirm significant association with anemia.

**Result:** The magnitude of anemia in East African countries was found to be 36.5% [95% confidence interval (CI): 35.55%, 36.75%]. Besides, as for the generalized linear mixed-effect model, age, educational status, working status, country of residence, wealth index, antenatal care service, place of delivery, history of using family planning in a health facility, current pregnancy, and visited by fieldworker in the last 12 months were factors that have a significant association with anemia in lactating mothers.

**Conclusion:** In East Africa, more than one-third of lactating mothers have anemia. The odds of anemia were significantly low among young mothers (15–34), who had primary education, were working, country of residence, and higher wealth index (middle and high). In addition, the likelihood of anemia was also low among lactating mothers who had antenatal care, used family planning, delivered at a health facility, were pregnant during the survey, and visited by fieldworkers. Therefore, promoting maternal care services (family planning, Antenatal Care (ANC), and delivery at health facilities) and a field visit by health extension workers are strongly recommended.

## Background

Anemia is a disorder defined as a reduced absolute number of circulating red blood cells, indicated by a low serum hemoglobin concentration (1). It occurs when a low number of red blood cells in the circulatory system leaves the oxygen-carrying capacity insufficient to meet physiological needs (2). Besides, it is one of the major health problems which is estimated to affect nearly 2 billion people all over the world (3, 4).

Anemia is widely spread and prevalent in developing countries than in developed countries (3–6) where Sub-Saharan Africa and Southeast Asia bear the highest- burden (7). Globally, 54.1% of anemia cases contribute to mild, 42.5% moderate, and 3.4% severe problems in all age groups of the population of the world (8, 9). The magnitude of anemia among lactating mothers is 52.5% in South Asia (4), 60.3% in Myanmar, 20% in Nepal, 63% in India, and 28.3% in Ethiopia (4, 6, 10, 11).

A wide variety of factors attribute to anemia and most of them are coincident. Iron deficiency is the most significant contributor to the occurrence of anemia, which accounts for 50% of the cases. Whereas vitamin deficiencies (folate and vitamin B12), infections, and hemoglobinopathies contribute to the rest of the cases of anemia worldwide (1–3, 5, 6). Lactating women, women of reproductive age, adolescent girls, pregnant women, newborn infants, and young children are the major risk groups for anemia (3, 12).

Lactating mothers commonly acquire anemia during their pregnancy (6). Lactating mothers are vulnerable to anemia morbidity due to their susceptibility to iron depletion during pregnancy and bad consequences of blood loss during their childbirth (13). Anemia in lactating women is also associated with a history of abortion, residence, history of malaria, and tea consumption (6, 12, 13). Prominently, lack of formal education, rural residency, higher parity, lower antenatal care visits, lack of family planning utilization, underweight, lower dietary diversity, food insecurity, and malaria infection were factors associated with higher odds of developing anemia while taking iron supplementation, being employed women and rich wealth quintile were significantly associated with lower risk of anemia (14).

Despite the high prevalence of anemia among lactating mothers particularly in developing countries, information on the magnitude of anemia and its determinant factors among lactating mothers remains unclear. In addition, few studies conducted were at the country level and different parts of countries. Therefore, the purpose of this study is to determine the magnitude of anemia among lactating mothers and its determinant factors in East Africa. As a result, the finding from this study will be helpful for health planners, decision-makers, and health professionals in understanding the burden of anemia among lactating mothers and in designing evidence-based interventions in the region.

## Methods

### Study Setting

According to the classification of the United Nations (UN), the African continent is subdivided into five regions. Among these regions, East Africa is one of the largest regions that includes 19 countries (Burundi, Comoros, Djibouti, Ethiopia, Eritrea, Kenya, Madagascar, Malawi, Mauritius, Mozambique, Reunion, Rwanda, Seychelles, Somalia, Somaliland, Tanzania, Uganda, Zambia, and Zimbabwe). The current study was conducted based on the Demographic and Health Surveys (DHS) data. From these 19 East African countries, six countries (Djibouti, Somalia, Somaliland, Seychelles, Mauritius, and Reunion) have no DHS data. Among the rest 13 countries that have DHS data, two countries have DHS data that was conducted before 2010 (Eritrea-2002 and Madagascar-2008) and two countries (Kenya and Comoros) did not assess anemia levels. Therefore, we included the nine countries that conducted DHS after 2010.

### Data Source

The data of these nine East African countries were taken from the DHS program official database www.measuredhs.com after authorization was approved as a result of an online request made by clarifying the aim of this study. DHS is a nationally representative household survey that contains data from a wide variety of population, health, and nutrition tracking and effect assessment measures with face-to-face interviews of women aged 15–49. It also adopts standardized methods involving uniform questionnaires, manuals, and field procedures. Three types of sampling—stratified, multistage, and random—are used in the survey. In each country, information was obtained from qualified women aged 15–49 years. Detailed survey techniques and methods of sampling used to collect data have been reported elsewhere (13). Dependent and independent variables were extracted from the individual record (IR file) data set. From nine East African countries, a total weighted sample of 25,425 lactating mothers was included in the study.

### Study Variables

The dependent variable of the present study was anemia status. The outcome variable was binary and it was coded as 1 if the lactating mother is anemic and 0 if the lactating mother is non-anemic. Based on different literatures, age, marital status, educational level, current work status, place of residence, wealth index, sex of head of household, age of head of household, cigarette smoking, media exposure, visited by fieldworker in the last 12 months, visited a health facility in the last 12 months, parity, antenatal care, place of delivery, current pregnancy, and use of family planning were considered as independent variables.

### Data Processing and Statistical Analysis

Data processing and analysis were done using STATA 14 software. The data were weighted using sampling weight, primary sampling unit, and strata before any statistical analysis was made in an attempt to restore the representativeness of the survey and to tell the STATA to take into account the sampling design when calculating SEs to get reliable statistical estimates. In addition, cross-tabulations and summary statistics were conducted to describe the study population.

Since the DHS data have a hierarchical nature, lactating mothers within a cluster may be more similar to each other than lactating mothers in the other clusters. Due to this, the assumption of independence of observations and equal variance across clusters might be violated. Therefore, an advanced statistical model is necessary to take into account the between cluster variability to get a reliable SE and unbiased estimate. Accordingly, both fixed and mixed effects were fitted. Model comparison was done based on the Akaike and Bayesian information criteria (AIC and BIC). The intra-cluster correlation coefficient (ICC) was also computed to measure the variation between clusters. A mixed-effect model [Generalized Linear Mixed Model (GLMM)] with the lowest Information Criteria (AIC and BIC) was selected (Table 1). Variables with a *p* ≤ 0.05 were declared as significant determinants of anemia.

**Table 1:** Model comparison between fixed effect and mixed effect logistic regression.

**Proposed model**	**AIC value**	**BIC value**	**ICC (95% CI)**
Fixed effect logistic regression	30514.99	30847.19	Not applicable
Mixed effect logistic regression	30462.71	30803.01	0.17 (0.12, 0.24)

*AIC, Akaike information criterion; BIC, Bayesian information criterion; CI, confidence interval; ICC, intra-cluster correlation coefficient*.

## Results

### Sociodemographic Characteristics

In this study, a total weighted sample of 25,425 lactating mothers from nine East African countries was included in the analysis. The larger proportion (18.32%) of the participants were from Ethiopia. The majority of lactating mothers (24.99%) included in the study were in the age range of 20–24 years. Most (86.04%) of the study participants were married at the time of the survey. More than half (50.62%) of the study participants attended primary level of education, and 55.05% were working at the time of the survey.

More than three-fourth (81.03%) of the lactating mothers were from rural areas, and the majority (47.53%) of them were from households with the poor wealth quintile. Regarding the age and sex of the household head, about 18.05% and more than three-fourth (78.22%) of respondents were in the age range of 25–29 years and from male-headed households, respectively (Table 2).

**Table 2:** Sociodemographic characteristics of respondents for the study on magnitudes of anemia and its determinant factors among lactating mothers in East African countries, 2021.

**Variables**	**Anemia**		**Weighted** **frequency**	**Percent**
	**Yes**	**No**		
**Age**				
15–19	1,093	1,551	2,644	10.40
20–24	2,252	4,103	6,355	24.99
25–29	2,147	4,186	6,333	24.91
30–34	1,761	3,236	4,997	19.66
35–39	1,241	2,084	3,325	13.08
40–44	547	882	1,429	5.62
45–49	150	192	342	1.34
**Marital status**				
Never married	603	971	1,574	6.19
Currently married	7,763	14,113	21,876	86.04
Formerly/ever married	825	1,150	1,975	7.77
**Educational level**				
Uneducated	3,129	4,421	7,550	29.70
Primary	4,569	8,302	12,871	50.62
Secondary	1,360	3,137	4,497	17.69
Higher	133	374	507	1.99
**Currently working**				
No	4,119	7,308	11,427	44.95
Yes	5,072	8,926	13,998	55.05
**Place of residence**				
Urban	1,637	3,185	4,822	18.97
Rural	7,554	13,049	20,603	81.03
**Country**				
Burundi	1,329	1,626	2,955	11.63
Ethiopia	1,316	3,341	4,657	18.32
Malawi	599	1,436	2,035	8.01
Mozambique	2,230	1,971	4,201	16.52
Rwanda	366	1,527	1,893	7.44
Tanzania	1,618	1,878	3,496	13.75
Uganda	522	1,023	1,545	6.08
Zambia	830	2,176	3,006	11.82
Zimbabwe	381	1,256	1,637	6.44
**Wealth index**				
Poor	4,761	7,323	12,084	47.53
Middle	1,815	3,311	5,126	20.16
Rich	2,615	5,600	8,215	32.31
**Sex of head of household**				
Male	7,011	12,877	19,888	78.22
Female	2,180	3,357	5,537	20.99
**Age of head of household**				
15–19	103	119	222	0.88
20–24	804	1,373	2,177	8.56
25–29	1,614	2,975	4,589	18.05
30–34	1,628	3,325	4,953	19.48
35–39	1,525	2,706	4,231	16.64
40–44	1,044	1,905	2,949	11.60
45–49	813	1,194	2,007	7.89
>49	1,660	2,637	4,297	16.90

### Behavioral and Obstetric Factors

Almost all (99.38%) of the study participants were no-smokers, and more than half (59.44%) had media exposure. About 74.57% of the study participants visited health facilities, and only 17.07% were visited by fieldworkers in the past 12 months preceding the survey. More than three-fourth (77.45%) of the pregnant women were multiparous and the majority (91.06%) had an ANC follow-up. Around 38.08% of the pregnant women reported that they used family planning and more than two-thirds (70.46%) delivered at health facilities (Table 3).

**Table 3:** Responses on behavioral and obstetric factors for the study on prevalence and associated factors of anemia among lactating mothers in East African countries, 2021.

**Variables**	**Anemia**		**Weighted** **frequency**	**Percent**
	**Yes**	**No**		
**Cigarette smoking**				
No	9,131	16,136	25,267	99.38
Yes	60	98	158	0.62
**Media exposure**				
No	3,820	6,493	10,313	40.56
Yes	5,371	9,741	15,112	59.44
**Visited by field worker in last 12 months**				
No	7,948	13,137	21,085	82.93
Yes	1,243	3,097	4,340	17.07
**Visited health facility last 12 months**				
No	2,292	4,173	6,465	25.43
Yes	6,899	12,061	18,960	74.57
**Parity**				
Prim-Para	2,081	3,653	5,734	22.55
Multipara	7,110	12,581	19,691	77.45
**Antenatal care**				
No	876	1,396	2,272	8.94
Yes	8,315	14,838	23,153	91.06
**Place of delivery**				
Home	3,098	4,425	7,523	29.59
Health facility	6,093	11,809	17,902	70.41
**Currently pregnant**				
No	16,003	9,073	25,076	98.63
Yes	118	231	349	1.37
**Family planning used**				
No	6,710	9,033	15,743	61.92
Yes	2,481	7,201	9,682	38.08

### Magnitudes of Anemia Among Lactating Mothers

**Figure 1:**
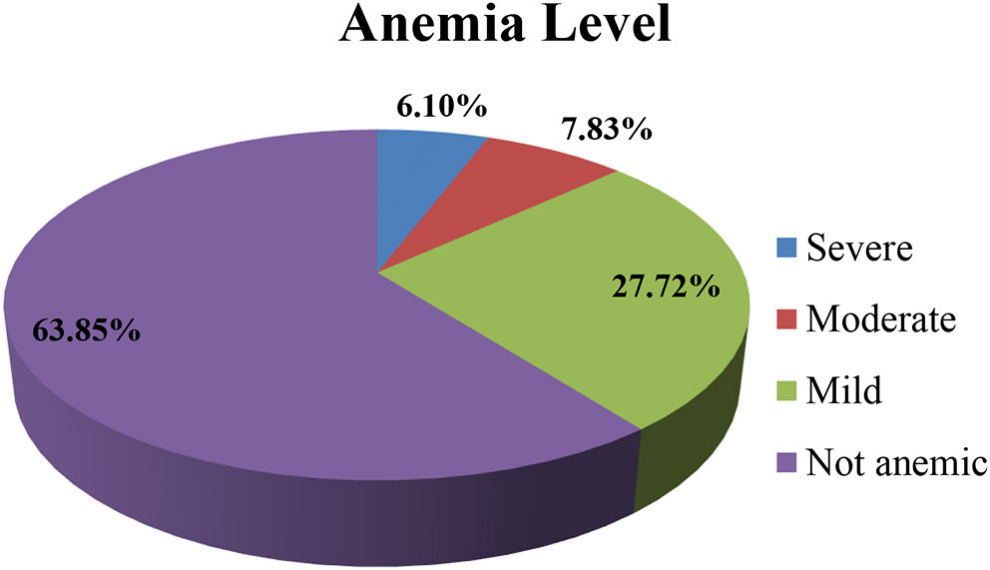
Magnitudes of anemia among lactating mothers in East African countries, 2021.

The overall prevalence of anemia among lactating mothers in East African countries was 36.15% [95% confidence interval (CI): 35.55, 36.75%]. Regarding the severity of anemia among lactating mothers, 6.10% had a severe form of anemia, and the majority (27.72%) and 7.83% had mild and moderate anemia, respectively (Figure 1). The highest prevalence of anemia was reported among lactating mothers from Mozambique (53.08%) followed by those from Tanzania (46.28%), whereas the lowest prevalence was from Rwanda (19.33%). The prevalence of anemia was highest (43.86%) among lactating mothers in the age group of 45–49 years and lowest (33.90%) in the age group of 25–29 years (Table 2).

### Factors Associated With Anemia Among Lactating Mothers

Age of mother, educational status, working status, country of residence, wealth index, being visited by fieldworker within 12 months, ANC visit, being pregnant, place of delivery, and family planning usage were significant predictors of anemia among lactating mothers (Table 4).

**Table 4:** Bi-variable and multi-variable mixed effect GLM analysis of anemia among lactating mothers in East African countries, 2021.

**Variables**	**Anemia**		**Odds Ratio [95% CI]**		***P*-value**
	**Yes**	**No**	**COR**	**AOR**	
**Age**					
15–19	1,093	1,551	1	1	
20–24	2,252	4,103	0.79 [0.71, 0.88]	**0.86 [0.76, 0.97]**	**0.012**
25–29	2,147	4,186	0.77 [0.70, 0.86]	**0.84 [0.73, 0.96]**	**0.013**
30–34	1,761	3,236	0.78 [0.70, 0.87]	**0.84 [0.73, 0.98]**	**0.024**
35–39	1,241	2,084	0.90 [0.80, 1.01]	0.91 [0.74, 1.06]	0.213
40–44	547	882	0.90 [0.77, 1.05]	0.85 [0.70, 1.01]	0.071
45–49	150	192	1.06 [0.82, 1.39]	0.90 [0.68, 1.19]	0.464
**Marital status**					
Currently married	7,763	14,113	1	1	
Never married	603	971	1.22 [1.08, 1.37]	1.04 [0.91, 1.20]	0.506
Formerly/ever married	825	1,150	1.21 [1.08, 1.35]	1.03 [0.92, 1.16]	0.621
**Educational level**					
Uneducated	3,129	4,421	1	1	
Primary	4,569	8,302	0.75 [0.70, 0.80]	**0.87 [0.80, 0.94]**	** <0.001**
Secondary	1,360	3,137	0.64 [0.59, 0.71]	0.90 [0.80, 1.01]	0.055
Higher	133	374	0.60 [0.48, 0.75]	1.08 [0.86, 1.36]	0.483
**Currently working**					
No	4,119	7,308	1	1	
Yes	5,072	8,926	0.89 [0.83, 0.95]	**0.89 [0.83, 0.95]**	**0.001**
**Place of residence**					
Urban	1,637	3,185	1	1	
Rural	7,554	13,049	1.25 [1.14, 1.36]	1.03 [0.93, 1.13]	0.575
**Country**					
Ethiopia	1,316	3,341	1	1	1
Burundi	1,329	1,626	1.65 [1.44, 1.90]	**2.02 [1.73, 2.36]**	** <0.001**
Malawi	599	1,436	0.95 [0.82, 1.09]	**1.37 [1.16, 1.60]**	** <0.001**
Mozambique	2,230	1,971	2.44 [2.14, 2.78]	**2.47 [2.15, 2.84]**	** <0.001**
Rwanda	366	1,527	0.48 [0.40, 0.56]	**0.68 [0.57, 0.82]**	** <0.001**
Tanzania	1,618	1,878	1.97 [1.73, 2.25]	**2.44 [2.10, 2.82]**	** <0.001**
Uganda	522	1,023	1.13 [0.97, 1.32]	**1.40 [1.18, 1.66]**	** <0.001**
Zambia	830	2,176	0.84 [0.73, 0.97]	1.04 [0.89, 1.21]	0.616
Zimbabwe	381	1,256	0.68 [0.58, 0.81]	1.01 [0.84, 1.21]	0.943
**Wealth index**					
Poor	4,761	7,323	1	1	
Middle	1,815	3,311	0.87 [0.80, 0.94]	**0.87 [0.80, 0.95]**	**0.001**
Rich	2,615	5,600	0.75 [0.70, 0.81]	**0.80 [0.74, 0.88]**	** <0.001**
**Sex of head of household**					
Male	7,011	12,877	1	1	
Female	2,180	3,357	1.14 [1.06, 1.22]	1.06 [0.98, 1.14]	0.165
**Age of head of household**					
15–19	103	119	1	1	
20–24	804	1,373	0.77 [0.57, 1.05]	0.95 [0.70, 1.30]	0.756
25–29	1,614	2,975	0.74 [0.55, 1.01]	0.99 [0.73, 1.35]	0.958
30–34	1,628	3,325	0.71 [0.53, 0.96]	0.96 [0.71, 1.31]	0.812
35–39	1,525	2,706	0.79 [0.58, 1.06]	0.99 [0.72, 1.35]	0.941
40–44	1,044	1,905	0.78 [0.58, 1.06]	0.96 [0.71, 1.31]	0.806
45–49	813	1,194	0.98 [0.72, 1.33]	1.15 [0.83, 1.58]	0.400
>49	1,660	2,637	0.95 [0.70, 1.28]	1.10 [0.81, 1.50]	0.535
**Cigarette smoking**					
No	9,131	16,136	1	1	
Yes	60	98	1.173 [0.82, 1.67]	1.08 [0.76, 1.53]	0.668
**Media exposure**					
No	3,820	6,493	1	1	
Yes	5,371	9,741	0.85 [0.794, 0.90]	0.94 [0.89, 1.01]	0.106
**Visited by fieldworker in last 12 months**					
No	7,948	13,137	1	1	
Yes	1,243	3,097	0.75 [0.69, 0.82]	**0.91 [0.84, 0.99]**	**0.037**
**Visited health facility last 12 months**					
No	2,292	4,173	1	1	
Yes	6,899	12,061	0.97 [0.91, 1.04]	1.01 [0.93, 1.08]	0.929
**Parity**					
Prim-Para	2,081	3,653	1	1	
Multipara	7,110	12,581	0.99 [0.92, 1.06]	1.01 [0.92, 1.11]	0.772
**Antenatal care**					
No	876	1,396	1	1	
Yes	8,315	14,838	0.76 [0.67, 0.85]	**0.85 [0.75, 0.97]**	**0.017**
**Place of delivery**					
Home	3,098	4,425	1	1	
Health facility	6,093	11,809	0.72 [0.67, 0.78]	**0.90 [0.83, 0.98]**	**0.012**
**Currently pregnant**					
No	16,003	9,073	1	1	
Yes	118	231	0.87 [0.67, 1.12]	**0.77 [0.59, 0.99]**	**0.038**
**Family planning used**					
No	6,710	9,033			
Yes	2,481	7,201	0.49 [0.46, 0.52]	**0.60 [0.56, 0.65]**	** <0.001**

*AOR, Adjusted Odd Ratio; CI, confidence interval; COR, Crude Odd Ratio. The bold values shows variables that have a significant association with anemia*.

The odds of having anemia decreased by 14% [adjusted odds ratio (AOR) = 0.86, 95% CI: 0.76, 0.97], 16% (AOR = 0.84, 95% CI: 0.73, 0.96), and 16% (AOR = 0.84, 95% CI: 0.73, 0.98) among lactating mothers aged 20–24, 25–29, and 30–34 years, respectively, as compared to those aged 15–19 years. The likelihood of being anemic was 13% (AOR = 0.87, 95% CI: 0.80, 0.94) lower among lactating mothers with a primary level of education compared to those who are uneducated. Anemia prevalence was lower by 13% (AOR = 0.87, 95% CI: 0.80, 0.94) among pregnant women who were working at the time of the survey as compared to those who were not working at the time of the survey.

The odds of having anemia were 2.02 (AOR = 2.02, 95% CI: 1.73, 2.36), 1.37 (AOR = 1.37, 95% CI: 1.16, 1.60), 2.47 (AOR = 2.47, 95% CI: 2.15, 2.84), 2.44 (AOR = 2.44, 95% CI: 2.10, 2.82), and 1.40 (AOR = 1.40, 95% CI: 1.18, 1.66) times higher among lactating mothers from Burundi, Malawi, Mozambique, Tanzania, and Uganda, respectively, as compared to those from Ethiopia. On the other hand, the likelihood of having anemia decreased by 32% (AOR = 0.68, 95% CI: 0.57, 0.82) among lactating mothers from Rwanda as compared to mothers in Ethiopia.

The odds of having anemia decreased by 13% (AOR = 0.87, 95% CI: 0.80, 0.95) and 20% (AOR = 0.80, 95% CI: 0.74, 0.88) among pregnant women from households in the middle and rich wealth quintiles, respectively, as compared to those in poor wealth quintiles. The likelihood of lactating mothers was anemic and lower by 9% (AOR = 0.91, 95% CI: 0.84, 0.99) among mothers who were visited by fieldworkers as compared to those not visited by a fieldworker.

Having an ANC visit decreased the odds of developing anemia among lactating mothers by 15% (AOR = 0.85, 95% CI: 0.75, 0.97). Similarly, the odds of being anemic decreased by 10% (AOR = 0.90, 95% CI: 0.83, 0.98) among lactating mothers who delivered at the health facility as compared to those who delivered at home.

Lactating mothers who were pregnant at the time of the survey had 23% lower odds of developing anemia (AOR = 0.77, 95% CI: 0.59, 0.99) as compared to those who were not pregnant at the time of the survey. Likewise, family planning usage was associated with a 40% decrease in the odds of having anemia among lactating mothers as compared to mothers who did not use family planning (Table 3).

## Discussion

Anemia in lactating mothers is an overlooked public health issue that affects both the mother and the newborn (15). Thus, we examined the magnitude and determinant factors of anemia among lactating women in East Africa. Accordingly, the magnitude of anemia in East African countries was found to be 36.5%. In relation to the generalized linear mixed-effect model, age, educational status, working status, country of residence, wealth index, antenatal care service, place of delivery, history of using family planning, delivery at a health facility, being pregnant, and visited by fieldworkers in the last 12 months were factors that have a significant association with anemia in lactating mothers.

In this study, the prevalence of anemia among lactating mothers in East African countries was 36.5%, which is comparable to the study done in India (15). In addition, the prevalence of anemia observed among lactating mothers in this study was lower than the prevalence in studies conducted in Vietnam and Myanmar (11, 16). This might be because the mothers near and after delivery get enough maternity leave and consume animal products based on the culture and custom of their countries. The highest prevalence of anemia among East African countries was observed in Mozambique (53.08%), whereas the lowest was in Rwanda.

In the present study, the odds of having anemia were low among lactating mothers aged 20–34 years as compared to those aged 15–19 years. This is in-line with different studies that indicate the risk of anemia is greater in the later age group (17). Furthermore, in this study, the risk of anemia is lower by 13% in lactating mothers with education which is in-line with the study done in India (2).

On the other hand, the odds of having anemia are decreased as the wealth quantile is increased. This is in-line with the study done in Myanmar (11), and this might be because those lactating mothers with better socioeconomic status may access a balanced diet and buy a variety of iron-containing foods that help decrease the anemia incidence. Likewise, the likelihood of having anemia decreased among the lactating mothers who were visited by a fieldworker. This may imply that strengthening the fieldworker visits might decrease the anemia prevalence.

Moreover, lactating mothers with ANC follow-up had a decreased odd of having anemia. This is comparable with the study done in India (2). This could be because iron supplementation during pregnancy was provided to those who had ANC visits and decreased the prevalence of anemia. Besides, the current study documented that the odds of anemia among lactating mothers who delivered at the health facility were low as compared to their counterparts. This finding is in agreement with studies done in India (2), and this might be because the risk of hemorrhage among those lactating mothers who delivered at health institutions is lower. If it is happening in the context of skilled delivery, it can be easily managed, and this may contribute to the decrement of anemia.

Additionally, lactating mothers who were pregnant at the time of the survey had lower odds of developing anemia, which contradicts the study done in Ethiopia (18). This might be due to the ever-increasing coverage of maternal health services for pregnant mothers.

Furthermore, the odds of anemia in lactating mothers decreased among those who used family planning. This finding is in-line with studies done in Ethiopia (19, 20). This might be explained by the rationale that those lactating mothers who use family planning may have frequent contact with the health professional and in the process they may get additional nutritional advice that may contribute to decreasing anemia.

The current study has its strengths and limitations. The first strength is attributed to the use of a large sample size and nationally representative data set of each included country. Second, by considering the clustered nature of the data, the advanced model was applied. On the other hand, coming to the limitations of the present study, the findings might not be representative of all the East African countries, for some countries have no DHS program, some did not assess anemia level, and some have old DHS data.

## Conclusion

In East Africa, more than one-third of lactating mothers have anemia. The odds of anemia were significantly low among mothers aged 15–34 years, who had primary education, were working, country of residence, and had higher wealth index (middle and high). In addition, the likelihood of anemia was also low among lactating mothers who had antenatal care, used family planning, delivered in the health facility, were pregnant during the survey, and visited by fieldworkers. Therefore, promoting maternal care services (family planning, ANC, and health facility delivery) and a field visit by health extension workers is strongly recommended.

## Data Availability Statement

The original contributions presented in the study are included in the article/supplementary material, further inquiries can be directed to the corresponding author.

## Ethics Statement

We requested Demography and Health Surveys (DHS) Program, and permission to download and use the data for this study from http://www.dhsprogram.com was approved by the 152155 reference number. What is more, there are no individual identifiers reported in any part of this manuscript. All the data management and analysis strictly followed the standard indicated in the manuals of DHS.

## Author Contributions

The conception of the study, design of the study, acquisition of data, analysis, and interpretation of data were conducted by BT. Data curation, drafting the article, revising it critically for intellectual content, validation, and final approval of the version to be published were done by AW, NB, and DE. All authors have read and approved the final manuscript.

## Conflict of Interest

The authors declare that the research was conducted in the absence of any commercial or financial relationships that could be construed as a potential conflict of interest.

## Publisher's Note

All claims expressed in this article are solely those of the authors and do not necessarily represent those of their affiliated organizations, or those of the publisher, the editors and the reviewers. Any product that may be evaluated in this article, or claim that may be made by its manufacturer, is not guaranteed or endorsed by the publisher.

## Abbreviations

AIC: Akaike information criterion
AOR: adjusted odds ratio
BIC: Bayesian information criterion
CI: confidence interval
DHS: Demography and Health Surveys
GLMM: generalized linear mixed models
ICC: intra-cluster correlation coefficient
IR: individual record
UN: United Nation.
